# Mandibular Odontogenic Fibromyxoma: A Case Report of Clinical and Radiographic Presentations

**DOI:** 10.7759/cureus.81981

**Published:** 2025-04-09

**Authors:** Sara Boukssim, Loubna Amminou, Nadia Cherradi, Hafsa El Ouazzani, Bassima Chami

**Affiliations:** 1 Department of Oral Surgery, Faculty of Dental Medicine, Mohammed V University, Rabat, MAR; 2 Department of Pathology HSR, Mohammed V University, Rabat, MAR

**Keywords:** benign jaw lesion, mandible, myxofibroma, odontogenic fibromyxoma, odontology

## Abstract

Odontogenic fibromyxoma (OFM) is an uncommon benign neoplasm originating from the odontogenic ectomesenchyme. It exhibits a slow-growing yet locally aggressive behavior, most frequently presenting in young adults, although it can occur at any age and shows no significant gender predilection. Despite its clinical relevance, current research provides limited information regarding the risk factors, causative agents, and molecular pathways that contribute to its development and aggressive behavior. We present a case of OFM located in the mandible of a 38-year-old African male who reported a painless swelling in the right mandible extending from the first premolar to the second premolar. The clinical examination revealed a limited, firm, painless vestibular swelling in the region of teeth 44 and 45. The affected teeth exhibited normal vitality and physiological mobility. Radiographic imaging demonstrated a multilocular radiolucency with a "soap bubble" appearance, without evidence of root resorption. Histopathological analysis confirmed the diagnosis of odontogenic fibromyxoma, showing a proliferation of fibroblasts and myofibroblasts within a loose, myxomatous stroma rich in acid mucopolysaccharides, with scattered stellate cells displaying minimal pleomorphism. This case underscores the importance of a comprehensive diagnostic approach that integrates clinical, radiological, and histopathological findings to accurately identify OFM and distinguish it from other similar jaw lesions. Although conservative treatments such as enucleation with curettage offer notable benefits, the ideal surgical approach and follow-up protocol remain subjects of ongoing investigation. A more profound understanding of OFM's underlying pathogenesis could lead to improved treatment outcomes and guide future research on targeted therapeutic strategies.

## Introduction

Odontogenic fibromyxoma (OFM) is a rare benign neoplasm originating from odontogenic ectomesenchyme [1]. It consists of 10% of all odontogenic tumors [1]. Even though OFM is a benign condition, it has a slow-growing but locally aggressive tendency [2]. The lesion can develop at any age, with reported cases ranging from one to 73 years and a mean age of 30. It shows no significant gender predilection [2]. OFM usually affects the mandible and is commonly identified after a painless swelling appears. This might result in tooth displacement or loosening [3,4]. On radiographic examination, OFM typically presents as a multilocular radiolucency with a characteristic "soap bubble" or "honeycomb" appearance [3]. Differentiating these features from other odontogenic lesions can be challenging. Histopathological examination reveals a proliferation of myofibroblasts and fibroblasts within a loose, myxoid stroma that is rich in acid mucopolysaccharides. Sporadic stellate-shaped cells may also be observed, typically exhibiting minimal pleomorphism [5].

While OFM typically affects younger individuals and is more frequently located in the posterior mandible, this case is noteworthy for its occurrence in the premolar region of a 38-year-old adult male of African origin, a relatively uncommon demographic and anatomical presentation. This case report has been reported in line with the SCARE (Surgical Case Report) criteria [6].

## Case presentation

**Figure 1:**
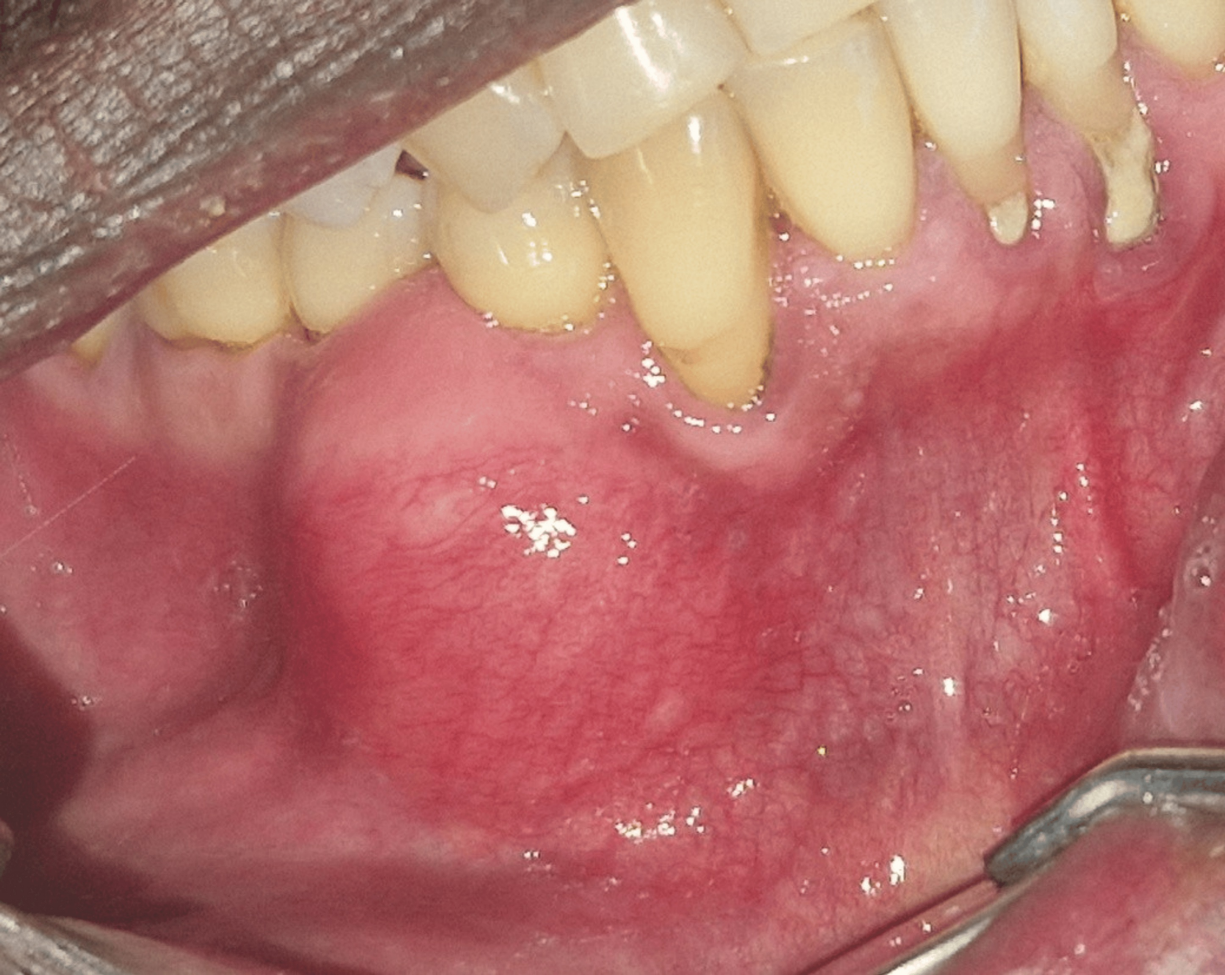
Intraoral view revealing a well-defined vestibular swelling covered with normal mucosa

A 38-year-old Guinean male patient presented at the oral surgery service with a chief complaint of right mandibular swelling that had evolved for three months. The patient's medical history revealed no general condition. An extraoral examination revealed no facial asymmetry, swelling, or deformity. An intraoral examination revealed a limited, firm, painless vestibular swelling involving the first and second right mandibular premolars. The swelling was covered with mucosa, which displayed a normal color. The examination also revealed compromised oral hygiene with evident tartaric deposits (Figure 1).

**Figure 2:**
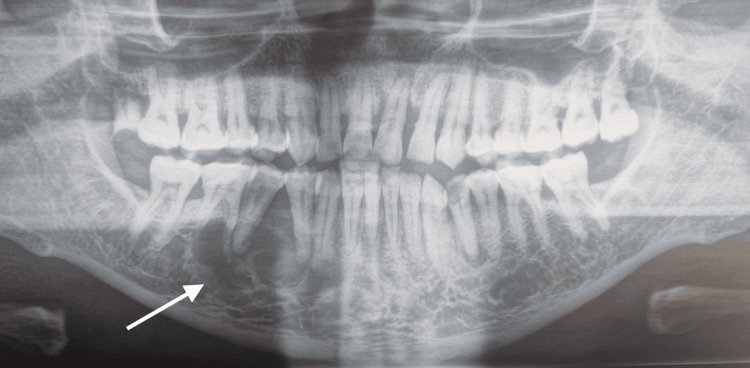
Orthopantomographic examination revealing a well-defined multilocular periapical lesion extending from tooth 44 to tooth 46

Teeth 44, 45, and 46 were vital and showed physiological mobility. The orthopantomographic examination revealed a well-defined multilocular periapical radiolucent image extending from tooth 43 to tooth 46 (Figure 2).

**Figure 3:**
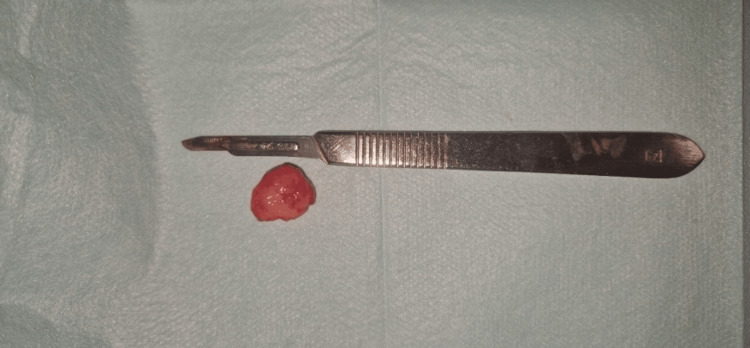
Macroscopic appearance of the OFM OFM: odontogenic fibromyxoma

The fine needle aspiration test was negative. The differential diagnosis included odontogenic keratocyst, ameloblastoma, central giant cell granuloma, and odontogenic myxoma. An excisional biopsy with curettage was performed due to the lesion's small size (Figure 3).

**Figure 4:**
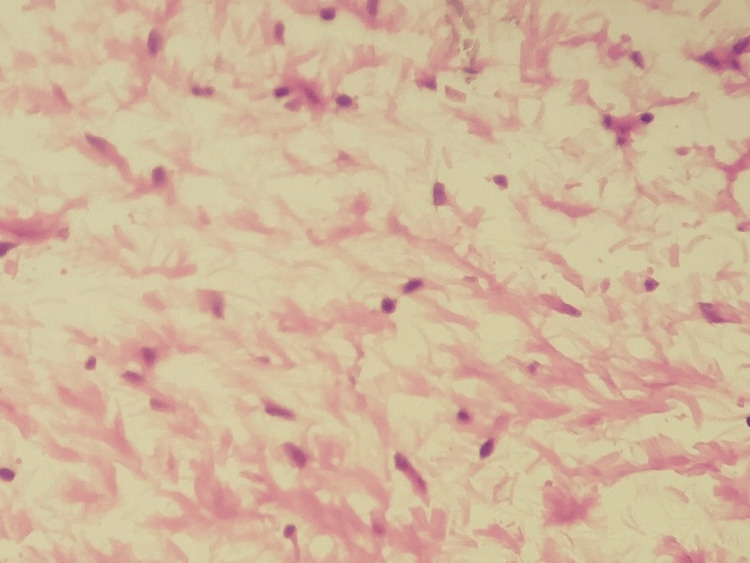
Histological aspect of the lesion showing spindle-shaped or stellate tumor cells, with no atypia and no mitotic figures (HE×40) HE×40: hematoxylin and eosin at 40× magnification

The histopathological examination revealed a benign, non-encapsulated mesenchymal proliferation with low cellular density. It consists of cells with a round nucleus, sometimes oval, sometimes spindle-shaped, with fine chromatin and poorly defined cytoplasm (Figure 4).

**Figure 5:**
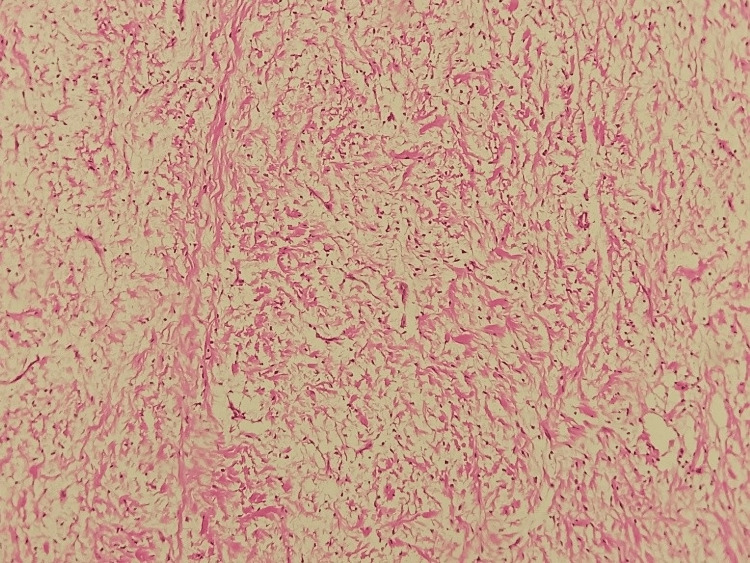
Microphotograph of an odontogenic myxoma, showing a benign tumor proliferation with low cellular density, developing within an abundant myxoid stroma (HE×10) HE×10: hematoxylin and eosin at 10× magnification

These cells were embedded in a loose, myxoid stroma that is abundantly vascularized, with occasional odontogenic remnants (Figure 5).

The results were suggestive of an odontogenic fibromyxoma. Following surgery, the patient returned to his home country and was referred to a local specialist for ongoing care. As a result, postoperative follow-up was not possible, and the long-term outcome remains unknown. However, the patient reported symptomatic relief prior to departure, with no immediate postoperative complications observed.

## Discussion

OFM is a rare benign tumor originating from the odontogenic ectomesenchyme [1]. Although it most commonly affects young adults in their second and third decades, OFM can occur at any age, with reported cases ranging from one to 73 years and no significant sex predilection [2,7]. However, some studies suggest that OFM may be more frequent in childhood compared to other aggressive odontogenic tumors [8]. The lesion can arise in both the maxilla and the mandible, with a higher incidence in the mandible, a finding that is consistent with the present case. OFM is characterized by gradual but locally invasive growth, accounting for less than 10% of all odontogenic tumors [4,7]. The pathophysiology, causal agents, and risk factors of OFM remain poorly understood [3]. Although some research has explored potential biomarkers for aggressive behavior, the exact biochemical mechanisms underlying the lesion's behavior are still unknown [3].

Clinically, patients typically present with a painless swelling, which may lead to local bone destruction, cortical expansion, soft tissue infiltration, root resorption, and tooth displacement [3]. In this case, the chief complaint was vestibular swelling; notably, the affected teeth remained vital with physiological mobility. According to Barros et al. [9], the radiographic characteristics of myxomas vary depending on the stage of tumor evolution. Advanced lesions tend to show extensive osteolysis, cortical expansion, and even infiltration into nearby soft tissues, while early lesions may appear as osteoporotic-like areas. Larger lesions are more likely to be multilocular, as demonstrated by Kaffe et al. [10] and Noffke et al. [4], who found a statistically significant association between lesion size and locularity.

The primary differential diagnoses include ameloblastoma, dentigerous cyst, odontogenic keratocyst, intraosseous hemangioma, aneurysmal bone cyst, and central giant cell granuloma (Table 1) [11,12].

**Table 1:** Clinicoradiological differential diagnosis of odontogenic myxoma

Condition	Clinical Features	Radiological Features
Odontogenic Myxoma	Painless, slow-growing, aggressive lesion, commonly in the posterior mandible, can cause tooth displacement and cortical expansion	Multilocular radiolucency with a "honeycomb" or "soap bubble" pattern and poorly defined borders, may cause root resorption
Ameloblastoma	Slow-growing, locally aggressive, often causes facial asymmetry, tooth displacement, and root resorption	Multilocular "soap bubble" or "honeycomb" appearance, well-defined borders, cortical expansion
Central Giant Cell Granuloma (CGCG)	Common in younger patients, can be asymptomatic or cause swelling and pain, and may lead to tooth mobility	Multilocular or unilocular radiolucency, often in the anterior mandible, well-defined but sometimes ill-defined borders
Odontogenic Keratocyst (OKC)	May be asymptomatic, can expand bone without cortical perforation, high recurrence rate	Unilocular or multilocular radiolucency with smooth, well-defined borders, often associated with impacted teeth
Hemangioma (Intraosseous)	May present with pulsatile swelling, pain, and bleeding on aspiration	Multilocular radiolucency with "sunburst" appearance, fine trabeculations, may have phleboliths
Aneurysmal Bone Cyst (ABC)	Rapidly growing, painful swelling, may cause cortical expansion and thinning, often in younger patients	Multilocular radiolucency with "blow-out" balloon-like expansion, thin cortices, may have fluid-fluid levels on CT

Histopathologically, OFM is characterized by a proliferation of fibroblasts and myofibroblasts embedded within a loose, myxomatous stroma rich in acidic mucopolysaccharides. Although minimal cellular pleomorphism may be present, it does not appear to correlate with recurrence rates. The relative proportions of collagen and mucoid material determine whether the lesion is classified as a myxofibroma or fibromyxoma. Additionally, the tumor exhibits spindle, hyaline, and stellate cell populations; the stellate cells typically express markers such as transferrin, S-100 protein, vimentin, and alpha-1 antitrypsin [5]. Grossly, these lesions present as well-delineated, semi-solid masses with a grayish-white to yellow appearance [2].

Surgical intervention is the treatment of choice for OFM; however, there is currently no consensus regarding the optimal extent of surgical margins. Given the rarity of the lesion, obtaining reliable prognostic data to compare various surgical approaches is challenging [13]. Although segmental resection is frequently performed, alternative conservative strategies, such as enucleation with adjunctive curettage or partial resection, have also been utilized, with reported recurrence rates being comparable between these modalities [3].

Conservative management, involving the enucleation of the lesion followed by curettage of the residual cavity, offers several advantages over more radical procedures. These benefits include reduced morbidity, decreased need for reconstructive surgery, shorter hospitalization, minimized interference with facial growth in pediatric patients, and overall lower treatment costs [13].

Nonetheless, some authors advocate for a more radical approach, such as en bloc resection, due to OFM's locally aggressive behavior, its potential for significant growth, and recurrence rates ranging from 10% to 43% [14,15].

Recurrence is frequently attributed to tumor infiltration into the cancellous bone beyond radiographically evident margins, particularly in the absence of a defined capsule [13]. The choice of conservative surgery is further supported by the lack of evidence for malignant transformation in OFM, along with reports of low recurrence rates in certain case series [14].

Regardless of the surgical method employed, vigilant postoperative monitoring is imperative. Although follow-up protocols for OFM are not well established in the literature, Dotta et al. [3] suggest a 10-year follow-up period with clinical and radiographic examinations conducted every six months, more frequently (every three months) during the first postoperative year, to ensure early detection of any recurrence.

What makes this case particularly rare is its localization in the mandibular premolar area and occurrence in a patient outside the most frequently affected age group. Such presentations are less commonly reported in the literature, thereby contributing to the broader clinical spectrum of OFM.

## Conclusions

This case highlights the importance of a thorough diagnostic approach in identifying OFM, particularly when it presents in atypical locations or age groups. The findings support the effectiveness of conservative management for small, well-defined lesions while also reinforcing the need for long-term follow-up due to the risk of recurrence. By documenting this rare presentation, this report contributes to the broader understanding of OFM's clinical variability and supports individualized treatment planning.
